# Supplementary material for: Pyruvate Dehydrogenase Kinase 1 inhibition mediated oxidative phosphorylation enhancement in cartilage promotes osteoarthritis progression
Source: BMC Musculoskelet Disord. 2023 Jul 20;24:597. doi: 10.1186/s12891-023-06585-6 (PMC10357736; doi:10.1186/s12891-023-06585-6)
Supplement: Supplementary file 1 — Additional file 1. [file 12891_2023_6585_MOESM1_ESM.zip › MF.Gsea.1653627316242/GOMF_RNA_POLYMERASE_II_SPECIFIC_DNA_BINDING_TRANSCRIPTION_FACTOR_BINDING.html]

Details for gene set GOMF\_RNA\_POLYMERASE\_II\_SPECIFIC\_DNA\_BINDING\_TRANSCRIPTION\_FACTOR\_BINDING[GSEA]

|  || Dataset | 55457\_collapsed\_to\_symbols.cls.cls #OA\_versus\_normal.cls.cls #OA\_versus\_normal\_repos |
| Phenotype | cls.cls#OA\_versus\_normal\_repos |
| Upregulated in class | normal |
| GeneSet | GOMF\_RNA\_POLYMERASE\_II\_SPECIFIC\_DNA\_BINDING\_TRANSCRIPTION\_FACTOR\_BINDING |
| Enrichment Score (ES) | -0.308214 |
| Normalized Enrichment Score (NES) | -1.5426195 |
| Nominal p-value | 0.0 |
| FDR q-value | 0.16125402 |
| FWER p-Value | 1.0 |
Table: GSEA Results Summary

  

Fig 1: Enrichment plot: GOMF\_RNA\_POLYMERASE\_II\_SPECIFIC\_DNA\_BINDING\_TRANSCRIPTION\_FACTOR\_BINDING      
 Profile of the Running ES Score & Positions of GeneSet Members on the Rank Ordered List

  

| SYMBOL | TITLE | RANK IN GENE LIST | RANK METRIC SCORE | RUNNING ES | CORE ENRICHMENT || 1 | TRIM68 | tripartite motif containing 68 [Source:HGNC Symbol;Acc:HGNC:21161] | 34 | 1.023 | 0.0070 | No |
| 2 | RNF14 | ring finger protein 14 [Source:HGNC Symbol;Acc:HGNC:10058] | 90 | 0.926 | 0.0115 | No |
| 3 | YWHAH | tyrosine 3-monooxygenase/tryptophan 5-monooxygenase activation protein eta [Source:HGNC Symbol;Acc:HGNC:12853] | 118 | 0.889 | 0.0178 | No |
| 4 | SMAD2 | SMAD family member 2 [Source:HGNC Symbol;Acc:HGNC:6768] | 275 | 0.768 | 0.0127 | No |
| 5 | ANXA4 | annexin A4 [Source:HGNC Symbol;Acc:HGNC:542] | 300 | 0.749 | 0.0179 | No |
| 6 | RBX1 | ring-box 1 [Source:HGNC Symbol;Acc:HGNC:9928] | 348 | 0.724 | 0.0211 | No |
| 7 | SLC30A9 | solute carrier family 30 member 9 [Source:HGNC Symbol;Acc:HGNC:1329] | 390 | 0.710 | 0.0246 | No |
| 8 | DNMT3A | DNA methyltransferase 3 alpha [Source:HGNC Symbol;Acc:HGNC:2978] | 406 | 0.704 | 0.0301 | No |
| 9 | BHLHE41 | basic helix-loop-helix family member e41 [Source:HGNC Symbol;Acc:HGNC:16617] | 417 | 0.700 | 0.0360 | No |
| 10 | TRIP6 | thyroid hormone receptor interactor 6 [Source:HGNC Symbol;Acc:HGNC:12311] | 524 | 0.650 | 0.0337 | No |
| 11 | NR0B2 | nuclear receptor subfamily 0 group B member 2 [Source:HGNC Symbol;Acc:HGNC:7961] | 573 | 0.632 | 0.0359 | No |
| 12 | KAT8 | lysine acetyltransferase 8 [Source:HGNC Symbol;Acc:HGNC:17933] | 595 | 0.625 | 0.0402 | No |
| 13 | FAF1 | Fas associated factor 1 [Source:HGNC Symbol;Acc:HGNC:3578] | 686 | 0.597 | 0.0387 | No |
| 14 | FBP1 | fructose-bisphosphatase 1 [Source:HGNC Symbol;Acc:HGNC:3606] | 772 | 0.576 | 0.0375 | No |
| 15 | CDKN2A | cyclin dependent kinase inhibitor 2A [Source:HGNC Symbol;Acc:HGNC:1787] | 799 | 0.570 | 0.0408 | No |
| 16 | GTF2A2 | general transcription factor IIA subunit 2 [Source:HGNC Symbol;Acc:HGNC:4647] | 899 | 0.543 | 0.0381 | No |
| 17 | ESR1 | estrogen receptor 1 [Source:HGNC Symbol;Acc:HGNC:3467] | 984 | 0.522 | 0.0364 | No |
| 18 | TRIM32 | tripartite motif containing 32 [Source:HGNC Symbol;Acc:HGNC:16380] | 993 | 0.519 | 0.0407 | No |
| 19 | NEUROD1 | neuronal differentiation 1 [Source:HGNC Symbol;Acc:HGNC:7762] | 1008 | 0.515 | 0.0445 | No |
| 20 | PRMT2 | protein arginine methyltransferase 2 [Source:HGNC Symbol;Acc:HGNC:5186] | 1171 | 0.482 | 0.0362 | No |
| 21 | TADA3 | transcriptional adaptor 3 [Source:HGNC Symbol;Acc:HGNC:19422] | 1295 | 0.459 | 0.0308 | No |
| 22 | GTF2B | general transcription factor IIB [Source:HGNC Symbol;Acc:HGNC:4648] | 1315 | 0.455 | 0.0336 | No |
| 23 | HDAC3 | histone deacetylase 3 [Source:HGNC Symbol;Acc:HGNC:4854] | 1325 | 0.454 | 0.0372 | No |
| 24 | WBP2 | WW domain binding protein 2 [Source:HGNC Symbol;Acc:HGNC:12738] | 1344 | 0.451 | 0.0401 | No |
| 25 | BUD31 | BUD31 homolog [Source:HGNC Symbol;Acc:HGNC:29629] | 1380 | 0.446 | 0.0416 | No |
| 26 | BBS4 | Bardet-Biedl syndrome 4 [Source:HGNC Symbol;Acc:HGNC:969] | 1404 | 0.442 | 0.0439 | No |
| 27 | PARK7 | Parkinsonism associated deglycase [Source:HGNC Symbol;Acc:HGNC:16369] | 1406 | 0.442 | 0.0481 | No |
| 28 | BCAS3 | BCAS3 microtubule associated cell migration factor [Source:HGNC Symbol;Acc:HGNC:14347] | 1471 | 0.433 | 0.0471 | No |
| 29 | BBS1 | Bardet-Biedl syndrome 1 [Source:HGNC Symbol;Acc:HGNC:966] | 1556 | 0.419 | 0.0444 | No |
| 30 | PCNA | proliferating cell nuclear antigen [Source:HGNC Symbol;Acc:HGNC:8729] | 1557 | 0.419 | 0.0484 | No |
| 31 | MLX | MAX dimerization protein MLX [Source:HGNC Symbol;Acc:HGNC:11645] | 1604 | 0.413 | 0.0487 | No |
| 32 | IFI27 | interferon alpha inducible protein 27 [Source:HGNC Symbol;Acc:HGNC:5397] | 1627 | 0.409 | 0.0508 | No |
| 33 | ZNHIT3 | zinc finger HIT-type containing 3 [Source:HGNC Symbol;Acc:HGNC:12309] | 1648 | 0.407 | 0.0531 | No |
| 34 | AIP | aryl hydrocarbon receptor interacting protein [Source:HGNC Symbol;Acc:HGNC:358] | 1649 | 0.406 | 0.0570 | No |
| 35 | CEBPA | CCAAT enhancer binding protein alpha [Source:HGNC Symbol;Acc:HGNC:1833] | 1750 | 0.392 | 0.0528 | No |
| 36 | CSNK2B | casein kinase 2 beta [Source:HGNC Symbol;Acc:HGNC:2460] | 1788 | 0.385 | 0.0535 | No |
| 37 | C1D | C1D nuclear receptor corepressor [Source:HGNC Symbol;Acc:HGNC:29911] | 1848 | 0.379 | 0.0524 | No |
| 38 | GBX2 | gastrulation brain homeobox 2 [Source:HGNC Symbol;Acc:HGNC:4186] | 1861 | 0.378 | 0.0550 | No |
| 39 | GTF2A1 | general transcription factor IIA subunit 1 [Source:HGNC Symbol;Acc:HGNC:4646] | 1936 | 0.368 | 0.0527 | No |
| 40 | NFE2L2 | "nuclear factor, erythroid 2 like 2 [Source:HGNC Symbol;Acc:HGNC:7782]" | 2034 | 0.357 | 0.0484 | No |
| 41 | HSPB1 | heat shock protein family B (small) member 1 [Source:HGNC Symbol;Acc:HGNC:5246] | 2105 | 0.349 | 0.0461 | No |
| 42 | LMO2 | LIM domain only 2 [Source:HGNC Symbol;Acc:HGNC:6642] | 2122 | 0.347 | 0.0482 | No |
| 43 | PITX1 | paired like homeodomain 1 [Source:HGNC Symbol;Acc:HGNC:9004] | 2263 | 0.331 | 0.0402 | No |
| 44 | CRKL | "CRK like proto-oncogene, adaptor protein [Source:HGNC Symbol;Acc:HGNC:2363]" | 2283 | 0.329 | 0.0418 | No |
| 45 | TRIB2 | tribbles pseudokinase 2 [Source:HGNC Symbol;Acc:HGNC:30809] | 2332 | 0.323 | 0.0411 | No |
| 46 | MAPK14 | mitogen-activated protein kinase 14 [Source:HGNC Symbol;Acc:HGNC:6876] | 2352 | 0.320 | 0.0426 | No |
| 47 | UBXN7 | UBX domain protein 7 [Source:HGNC Symbol;Acc:HGNC:29119] | 2354 | 0.320 | 0.0456 | No |
| 48 | ISL1 | ISL LIM homeobox 1 [Source:HGNC Symbol;Acc:HGNC:6132] | 2373 | 0.318 | 0.0472 | No |
| 49 | PKN1 | protein kinase N1 [Source:HGNC Symbol;Acc:HGNC:9405] | 2540 | 0.298 | 0.0368 | No |
| 50 | NCOR1 | nuclear receptor corepressor 1 [Source:HGNC Symbol;Acc:HGNC:7672] | 2549 | 0.297 | 0.0390 | No |
| 51 | MED12 | mediator complex subunit 12 [Source:HGNC Symbol;Acc:HGNC:11957] | 2588 | 0.292 | 0.0388 | No |
| 52 | BBS10 | Bardet-Biedl syndrome 10 [Source:HGNC Symbol;Acc:HGNC:26291] | 2688 | 0.280 | 0.0336 | No |
| 53 | NIF3L1 | NGG1 interacting factor 3 like 1 [Source:HGNC Symbol;Acc:HGNC:13390] | 2807 | 0.269 | 0.0267 | No |
| 54 | PADI2 | peptidyl arginine deiminase 2 [Source:HGNC Symbol;Acc:HGNC:18341] | 2810 | 0.269 | 0.0291 | No |
| 55 | KAT6B | lysine acetyltransferase 6B [Source:HGNC Symbol;Acc:HGNC:17582] | 2827 | 0.267 | 0.0304 | No |
| 56 | GATA4 | GATA binding protein 4 [Source:HGNC Symbol;Acc:HGNC:4173] | 3009 | 0.249 | 0.0184 | No |
| 57 | NFATC1 | nuclear factor of activated T cells 1 [Source:HGNC Symbol;Acc:HGNC:7775] | 3027 | 0.247 | 0.0194 | No |
| 58 | TAF7 | TATA-box binding protein associated factor 7 [Source:HGNC Symbol;Acc:HGNC:11541] | 3035 | 0.246 | 0.0212 | No |
| 59 | GAS2L1 | growth arrest specific 2 like 1 [Source:HGNC Symbol;Acc:HGNC:16955] | 3136 | 0.237 | 0.0155 | No |
| 60 | PTPRT | protein tyrosine phosphatase receptor type T [Source:HGNC Symbol;Acc:HGNC:9682] | 3157 | 0.234 | 0.0161 | No |
| 61 | PRAME | PRAME nuclear receptor transcriptional regulator [Source:HGNC Symbol;Acc:HGNC:9336] | 3235 | 0.227 | 0.0122 | No |
| 62 | MED24 | mediator complex subunit 24 [Source:HGNC Symbol;Acc:HGNC:22963] | 3246 | 0.226 | 0.0135 | No |
| 63 | DACT1 | dishevelled binding antagonist of beta catenin 1 [Source:HGNC Symbol;Acc:HGNC:17748] | 3275 | 0.223 | 0.0134 | No |
| 64 | LRIF1 | ligand dependent nuclear receptor interacting factor 1 [Source:HGNC Symbol;Acc:HGNC:30299] | 3284 | 0.222 | 0.0149 | No |
| 65 | ELK1 | ETS transcription factor ELK1 [Source:HGNC Symbol;Acc:HGNC:3321] | 3357 | 0.216 | 0.0113 | No |
| 66 | PARP1 | poly(ADP-ribose) polymerase 1 [Source:HGNC Symbol;Acc:HGNC:270] | 3410 | 0.210 | 0.0091 | No |
| 67 | MDFIC | MyoD family inhibitor domain containing [Source:HGNC Symbol;Acc:HGNC:28870] | 3419 | 0.209 | 0.0105 | No |
| 68 | ARNT2 | aryl hydrocarbon receptor nuclear translocator 2 [Source:HGNC Symbol;Acc:HGNC:16876] | 3512 | 0.201 | 0.0051 | No |
| 69 | VDR | vitamin D receptor [Source:HGNC Symbol;Acc:HGNC:12679] | 3571 | 0.197 | 0.0023 | No |
| 70 | TGFB1I1 | transforming growth factor beta 1 induced transcript 1 [Source:HGNC Symbol;Acc:HGNC:11767] | 3628 | 0.191 | -0.0003 | No |
| 71 | PRRX1 | paired related homeobox 1 [Source:HGNC Symbol;Acc:HGNC:9142] | 3657 | 0.189 | -0.0007 | No |
| 72 | RBBP8 | "RB binding protein 8, endonuclease [Source:HGNC Symbol;Acc:HGNC:9891]" | 3739 | 0.182 | -0.0054 | No |
| 73 | HMGN3 | high mobility group nucleosomal binding domain 3 [Source:HGNC Symbol;Acc:HGNC:12312] | 3818 | 0.174 | -0.0099 | No |
| 74 | CNOT1 | CCR4-NOT transcription complex subunit 1 [Source:HGNC Symbol;Acc:HGNC:7877] | 3842 | 0.172 | -0.0101 | No |
| 75 | ADD1 | adducin 1 [Source:HGNC Symbol;Acc:HGNC:243] | 3872 | 0.167 | -0.0108 | No |
| 76 | RELA | "RELA proto-oncogene, NF-kB subunit [Source:HGNC Symbol;Acc:HGNC:9955]" | 3942 | 0.161 | -0.0148 | No |
| 77 | NR1I2 | nuclear receptor subfamily 1 group I member 2 [Source:HGNC Symbol;Acc:HGNC:7968] | 3956 | 0.160 | -0.0143 | No |
| 78 | ZFPM2 | "zinc finger protein, FOG family member 2 [Source:HGNC Symbol;Acc:HGNC:16700]" | 3958 | 0.160 | -0.0129 | No |
| 79 | HEY2 | hes related family bHLH transcription factor with YRPW motif 2 [Source:HGNC Symbol;Acc:HGNC:4881] | 4058 | 0.150 | -0.0193 | No |
| 80 | SMAD3 | SMAD family member 3 [Source:HGNC Symbol;Acc:HGNC:6769] | 4061 | 0.150 | -0.0180 | No |
| 81 | KEAP1 | kelch like ECH associated protein 1 [Source:HGNC Symbol;Acc:HGNC:23177] | 4095 | 0.146 | -0.0193 | No |
| 82 | TRIP4 | thyroid hormone receptor interactor 4 [Source:HGNC Symbol;Acc:HGNC:12310] | 4105 | 0.146 | -0.0186 | No |
| 83 | MKKS | MKKS centrosomal shuttling protein [Source:HGNC Symbol;Acc:HGNC:7108] | 4182 | 0.140 | -0.0233 | No |
| 84 | WIPI1 | "WD repeat domain, phosphoinositide interacting 1 [Source:HGNC Symbol;Acc:HGNC:25471]" | 4188 | 0.139 | -0.0224 | No |
| 85 | MEF2C | myocyte enhancer factor 2C [Source:HGNC Symbol;Acc:HGNC:6996] | 4218 | 0.135 | -0.0234 | No |
| 86 | SMARCE1 | "SWI/SNF related, matrix associated, actin dependent regulator of chromatin, subfamily e, member 1 [Source:HGNC Symbol;Acc:HGNC:11109]" | 4255 | 0.132 | -0.0250 | No |
| 87 | TAF6 | TATA-box binding protein associated factor 6 [Source:HGNC Symbol;Acc:HGNC:11540] | 4260 | 0.132 | -0.0241 | No |
| 88 | NCOA2 | nuclear receptor coactivator 2 [Source:HGNC Symbol;Acc:HGNC:7669] | 4284 | 0.129 | -0.0246 | No |
| 89 | PROX1 | prospero homeobox 1 [Source:HGNC Symbol;Acc:HGNC:9459] | 4299 | 0.128 | -0.0245 | No |
| 90 | TCF21 | transcription factor 21 [Source:HGNC Symbol;Acc:HGNC:11632] | 4321 | 0.127 | -0.0250 | No |
| 91 | HDAC5 | histone deacetylase 5 [Source:HGNC Symbol;Acc:HGNC:14068] | 4336 | 0.125 | -0.0249 | No |
| 92 | CD34 | CD34 molecule [Source:HGNC Symbol;Acc:HGNC:1662] | 4341 | 0.125 | -0.0241 | No |
| 93 | PHB2 | prohibitin 2 [Source:HGNC Symbol;Acc:HGNC:30306] | 4354 | 0.124 | -0.0238 | No |
| 94 | STAT1 | signal transducer and activator of transcription 1 [Source:HGNC Symbol;Acc:HGNC:11362] | 4495 | 0.113 | -0.0339 | No |
| 95 | SETD3 | "SET domain containing 3, actin histidine methyltransferase [Source:HGNC Symbol;Acc:HGNC:20493]" | 4506 | 0.113 | -0.0336 | No |
| 96 | PRPF6 | pre-mRNA processing factor 6 [Source:HGNC Symbol;Acc:HGNC:15860] | 4567 | 0.108 | -0.0373 | No |
| 97 | FLT3 | fms related receptor tyrosine kinase 3 [Source:HGNC Symbol;Acc:HGNC:3765] | 4657 | 0.099 | -0.0435 | No |
| 98 | TBX5 | T-box transcription factor 5 [Source:HGNC Symbol;Acc:HGNC:11604] | 4748 | 0.091 | -0.0497 | No |
| 99 | TBX3 | T-box transcription factor 3 [Source:HGNC Symbol;Acc:HGNC:11602] | 4790 | 0.088 | -0.0522 | No |
| 100 | OASL | 2'-5'-oligoadenylate synthetase like [Source:HGNC Symbol;Acc:HGNC:8090] | 4812 | 0.086 | -0.0530 | No |
| 101 | WWP2 | WW domain containing E3 ubiquitin protein ligase 2 [Source:HGNC Symbol;Acc:HGNC:16804] | 4818 | 0.086 | -0.0526 | No |
| 102 | H2BC9 | H2B clustered histone 9 [Source:HGNC Symbol;Acc:HGNC:4755] | 4859 | 0.083 | -0.0550 | No |
| 103 | TP53BP1 | tumor protein p53 binding protein 1 [Source:HGNC Symbol;Acc:HGNC:11999] | 4946 | 0.076 | -0.0611 | No |
| 104 | PPID | peptidylprolyl isomerase D [Source:HGNC Symbol;Acc:HGNC:9257] | 5029 | 0.071 | -0.0669 | No |
| 105 | TCF7L2 | transcription factor 7 like 2 [Source:HGNC Symbol;Acc:HGNC:11641] | 5034 | 0.070 | -0.0666 | No |
| 106 | SMARCD3 | "SWI/SNF related, matrix associated, actin dependent regulator of chromatin, subfamily d, member 3 [Source:HGNC Symbol;Acc:HGNC:11108]" | 5036 | 0.070 | -0.0660 | No |
| 107 | CTBP2 | C-terminal binding protein 2 [Source:HGNC Symbol;Acc:HGNC:2495] | 5066 | 0.067 | -0.0676 | No |
| 108 | NSD1 | nuclear receptor binding SET domain protein 1 [Source:HGNC Symbol;Acc:HGNC:14234] | 5178 | 0.058 | -0.0759 | No |
| 109 | HDAC9 | histone deacetylase 9 [Source:HGNC Symbol;Acc:HGNC:14065] | 5183 | 0.058 | -0.0757 | No |
| 110 | TP73 | tumor protein p73 [Source:HGNC Symbol;Acc:HGNC:12003] | 5222 | 0.055 | -0.0782 | No |
| 111 | FOXC1 | forkhead box C1 [Source:HGNC Symbol;Acc:HGNC:3800] | 5227 | 0.054 | -0.0780 | No |
| 112 | MED17 | mediator complex subunit 17 [Source:HGNC Symbol;Acc:HGNC:2375] | 5257 | 0.052 | -0.0798 | No |
| 113 | TAL1 | "TAL bHLH transcription factor 1, erythroid differentiation factor [Source:HGNC Symbol;Acc:HGNC:11556]" | 5406 | 0.040 | -0.0912 | No |
| 114 | NR1H2 | nuclear receptor subfamily 1 group H member 2 [Source:HGNC Symbol;Acc:HGNC:7965] | 5413 | 0.039 | -0.0913 | No |
| 115 | SNW1 | SNW domain containing 1 [Source:HGNC Symbol;Acc:HGNC:16696] | 5472 | 0.035 | -0.0955 | No |
| 116 | TP53 | tumor protein p53 [Source:HGNC Symbol;Acc:HGNC:11998] | 5511 | 0.032 | -0.0983 | No |
| 117 | GATA6 | GATA binding protein 6 [Source:HGNC Symbol;Acc:HGNC:4174] | 5537 | 0.030 | -0.1000 | No |
| 118 | STAT5B | signal transducer and activator of transcription 5B [Source:HGNC Symbol;Acc:HGNC:11367] | 5554 | 0.028 | -0.1010 | No |
| 119 | GFI1B | growth factor independent 1B transcriptional repressor [Source:HGNC Symbol;Acc:HGNC:4238] | 5591 | 0.025 | -0.1036 | No |
| 120 | MEF2A | myocyte enhancer factor 2A [Source:HGNC Symbol;Acc:HGNC:6993] | 5628 | 0.022 | -0.1062 | No |
| 121 | SMARCA1 | "SWI/SNF related, matrix associated, actin dependent regulator of chromatin, subfamily a, member 1 [Source:HGNC Symbol;Acc:HGNC:11097]" | 5723 | 0.014 | -0.1136 | No |
| 122 | ARID1A | AT-rich interaction domain 1A [Source:HGNC Symbol;Acc:HGNC:11110] | 5735 | 0.013 | -0.1143 | No |
| 123 | KAT6A | lysine acetyltransferase 6A [Source:HGNC Symbol;Acc:HGNC:13013] | 5778 | 0.010 | -0.1176 | No |
| 124 | MED14 | mediator complex subunit 14 [Source:HGNC Symbol;Acc:HGNC:2370] | 5867 | 0.004 | -0.1245 | No |
| 125 | PPARG | peroxisome proliferator activated receptor gamma [Source:HGNC Symbol;Acc:HGNC:9236] | 6006 | -0.005 | -0.1354 | No |
| 126 | RB1 | RB transcriptional corepressor 1 [Source:HGNC Symbol;Acc:HGNC:9884] | 6013 | -0.006 | -0.1359 | No |
| 127 | FOXL2 | forkhead box L2 [Source:HGNC Symbol;Acc:HGNC:1092] | 6068 | -0.009 | -0.1401 | No |
| 128 | PPARGC1B | PPARG coactivator 1 beta [Source:HGNC Symbol;Acc:HGNC:30022] | 6184 | -0.019 | -0.1490 | No |
| 129 | ANKRD2 | ankyrin repeat domain 2 [Source:HGNC Symbol;Acc:HGNC:495] | 6186 | -0.019 | -0.1489 | No |
| 130 | MED16 | mediator complex subunit 16 [Source:HGNC Symbol;Acc:HGNC:17556] | 6240 | -0.023 | -0.1529 | No |
| 131 | ANKRD1 | ankyrin repeat domain 1 [Source:HGNC Symbol;Acc:HGNC:15819] | 6416 | -0.037 | -0.1664 | No |
| 132 | PRKCB | protein kinase C beta [Source:HGNC Symbol;Acc:HGNC:9395] | 6453 | -0.039 | -0.1689 | No |
| 133 | BBS7 | Bardet-Biedl syndrome 7 [Source:HGNC Symbol;Acc:HGNC:18758] | 6460 | -0.039 | -0.1690 | No |
| 134 | LHX3 | LIM homeobox 3 [Source:HGNC Symbol;Acc:HGNC:6595] | 6495 | -0.042 | -0.1713 | No |
| 135 | NCOR2 | nuclear receptor corepressor 2 [Source:HGNC Symbol;Acc:HGNC:7673] | 6497 | -0.042 | -0.1710 | No |
| 136 | CDK5RAP3 | CDK5 regulatory subunit associated protein 3 [Source:HGNC Symbol;Acc:HGNC:18673] | 6583 | -0.048 | -0.1773 | No |
| 137 | GTF2I | general transcription factor IIi [Source:HGNC Symbol;Acc:HGNC:4659] | 6588 | -0.049 | -0.1772 | No |
| 138 | BRMS1 | BRMS1 transcriptional repressor and anoikis regulator [Source:HGNC Symbol;Acc:HGNC:17262] | 6602 | -0.050 | -0.1777 | No |
| 139 | PSMA6 | proteasome 20S subunit alpha 6 [Source:HGNC Symbol;Acc:HGNC:9535] | 6721 | -0.057 | -0.1865 | No |
| 140 | MMS19 | "MMS19 homolog, cytosolic iron-sulfur assembly component [Source:HGNC Symbol;Acc:HGNC:13824]" | 6733 | -0.058 | -0.1869 | No |
| 141 | HMGA2 | high mobility group AT-hook 2 [Source:HGNC Symbol;Acc:HGNC:5009] | 6850 | -0.067 | -0.1954 | No |
| 142 | NR0B1 | nuclear receptor subfamily 0 group B member 1 [Source:HGNC Symbol;Acc:HGNC:7960] | 6885 | -0.070 | -0.1975 | No |
| 143 | SRC | "SRC proto-oncogene, non-receptor tyrosine kinase [Source:HGNC Symbol;Acc:HGNC:11283]" | 6940 | -0.075 | -0.2010 | No |
| 144 | JMJD1C | jumonji domain containing 1C [Source:HGNC Symbol;Acc:HGNC:12313] | 6989 | -0.079 | -0.2041 | No |
| 145 | SP3 | Sp3 transcription factor [Source:HGNC Symbol;Acc:HGNC:11208] | 7033 | -0.082 | -0.2067 | No |
| 146 | FOXP3 | forkhead box P3 [Source:HGNC Symbol;Acc:HGNC:6106] | 7035 | -0.082 | -0.2060 | No |
| 147 | CITED2 | Cbp/p300 interacting transactivator with Glu/Asp rich carboxy-terminal domain 2 [Source:HGNC Symbol;Acc:HGNC:1987] | 7100 | -0.087 | -0.2103 | No |
| 148 | HNF4A | hepatocyte nuclear factor 4 alpha [Source:HGNC Symbol;Acc:HGNC:5024] | 7160 | -0.093 | -0.2141 | No |
| 149 | NCOA6 | nuclear receptor coactivator 6 [Source:HGNC Symbol;Acc:HGNC:15936] | 7171 | -0.093 | -0.2140 | No |
| 150 | MED4 | mediator complex subunit 4 [Source:HGNC Symbol;Acc:HGNC:17903] | 7236 | -0.098 | -0.2181 | No |
| 151 | CALR | calreticulin [Source:HGNC Symbol;Acc:HGNC:1455] | 7291 | -0.102 | -0.2215 | No |
| 152 | RXRA | retinoid X receptor alpha [Source:HGNC Symbol;Acc:HGNC:10477] | 7381 | -0.109 | -0.2275 | No |
| 153 | DCAF13 | DDB1 and CUL4 associated factor 13 [Source:HGNC Symbol;Acc:HGNC:24535] | 7459 | -0.115 | -0.2325 | No |
| 154 | LEF1 | lymphoid enhancer binding factor 1 [Source:HGNC Symbol;Acc:HGNC:6551] | 7467 | -0.116 | -0.2320 | No |
| 155 | CREB1 | cAMP responsive element binding protein 1 [Source:HGNC Symbol;Acc:HGNC:2345] | 7470 | -0.116 | -0.2310 | No |
| 156 | TCF3 | transcription factor 3 [Source:HGNC Symbol;Acc:HGNC:11633] | 7521 | -0.120 | -0.2339 | No |
| 157 | TAF4B | TATA-box binding protein associated factor 4b [Source:HGNC Symbol;Acc:HGNC:11538] | 7596 | -0.127 | -0.2385 | No |
| 158 | NHLH2 | nescient helix-loop-helix 2 [Source:HGNC Symbol;Acc:HGNC:7818] | 7626 | -0.129 | -0.2396 | No |
| 159 | HCLS1 | hematopoietic cell-specific Lyn substrate 1 [Source:HGNC Symbol;Acc:HGNC:4844] | 7653 | -0.131 | -0.2404 | No |
| 160 | POU1F1 | POU class 1 homeobox 1 [Source:HGNC Symbol;Acc:HGNC:9210] | 7681 | -0.133 | -0.2413 | No |
| 161 | HIF1AN | hypoxia inducible factor 1 subunit alpha inhibitor [Source:HGNC Symbol;Acc:HGNC:17113] | 7815 | -0.142 | -0.2505 | No |
| 162 | MYOD1 | myogenic differentiation 1 [Source:HGNC Symbol;Acc:HGNC:7611] | 7839 | -0.143 | -0.2510 | No |
| 163 | HDAC1 | histone deacetylase 1 [Source:HGNC Symbol;Acc:HGNC:4852] | 7911 | -0.148 | -0.2552 | No |
| 164 | TFDP3 | transcription factor Dp family member 3 [Source:HGNC Symbol;Acc:HGNC:24603] | 7920 | -0.149 | -0.2544 | No |
| 165 | TAF4 | TATA-box binding protein associated factor 4 [Source:HGNC Symbol;Acc:HGNC:11537] | 7966 | -0.153 | -0.2565 | No |
| 166 | PITX2 | paired like homeodomain 2 [Source:HGNC Symbol;Acc:HGNC:9005] | 8137 | -0.166 | -0.2685 | No |
| 167 | ID4 | "inhibitor of DNA binding 4, HLH protein [Source:HGNC Symbol;Acc:HGNC:5363]" | 8174 | -0.169 | -0.2697 | No |
| 168 | HAND2 | heart and neural crest derivatives expressed 2 [Source:HGNC Symbol;Acc:HGNC:4808] | 8211 | -0.172 | -0.2709 | No |
| 169 | HEY1 | hes related family bHLH transcription factor with YRPW motif 1 [Source:HGNC Symbol;Acc:HGNC:4880] | 8213 | -0.172 | -0.2694 | No |
| 170 | AR | androgen receptor [Source:HGNC Symbol;Acc:HGNC:644] | 8217 | -0.173 | -0.2680 | No |
| 171 | KDM3A | lysine demethylase 3A [Source:HGNC Symbol;Acc:HGNC:20815] | 8264 | -0.176 | -0.2700 | No |
| 172 | TRIP12 | thyroid hormone receptor interactor 12 [Source:HGNC Symbol;Acc:HGNC:12306] | 8290 | -0.178 | -0.2702 | No |
| 173 | TP53BP2 | tumor protein p53 binding protein 2 [Source:HGNC Symbol;Acc:HGNC:12000] | 8336 | -0.181 | -0.2721 | No |
| 174 | HDAC4 | histone deacetylase 4 [Source:HGNC Symbol;Acc:HGNC:14063] | 8416 | -0.187 | -0.2766 | No |
| 175 | DUSP26 | dual specificity phosphatase 26 [Source:HGNC Symbol;Acc:HGNC:28161] | 8424 | -0.187 | -0.2754 | No |
| 176 | STAT3 | signal transducer and activator of transcription 3 [Source:HGNC Symbol;Acc:HGNC:11364] | 8453 | -0.190 | -0.2758 | No |
| 177 | NR1H4 | nuclear receptor subfamily 1 group H member 4 [Source:HGNC Symbol;Acc:HGNC:7967] | 8481 | -0.192 | -0.2761 | No |
| 178 | RNF6 | ring finger protein 6 [Source:HGNC Symbol;Acc:HGNC:10069] | 8493 | -0.193 | -0.2751 | No |
| 179 | LDB1 | LIM domain binding 1 [Source:HGNC Symbol;Acc:HGNC:6532] | 8522 | -0.195 | -0.2755 | No |
| 180 | NRIP1 | nuclear receptor interacting protein 1 [Source:HGNC Symbol;Acc:HGNC:8001] | 8524 | -0.195 | -0.2737 | No |
| 181 | CRY2 | cryptochrome circadian regulator 2 [Source:HGNC Symbol;Acc:HGNC:2385] | 8536 | -0.196 | -0.2727 | No |
| 182 | ATF2 | activating transcription factor 2 [Source:HGNC Symbol;Acc:HGNC:784] | 8592 | -0.201 | -0.2752 | No |
| 183 | KDM1A | lysine demethylase 1A [Source:HGNC Symbol;Acc:HGNC:29079] | 8608 | -0.202 | -0.2745 | No |
| 184 | ASXL1 | ASXL transcriptional regulator 1 [Source:HGNC Symbol;Acc:HGNC:18318] | 8610 | -0.202 | -0.2726 | No |
| 185 | CPNE1 | copine 1 [Source:HGNC Symbol;Acc:HGNC:2314] | 8642 | -0.204 | -0.2732 | No |
| 186 | AKAP8 | A-kinase anchoring protein 8 [Source:HGNC Symbol;Acc:HGNC:378] | 8659 | -0.205 | -0.2725 | No |
| 187 | RARG | retinoic acid receptor gamma [Source:HGNC Symbol;Acc:HGNC:9866] | 8700 | -0.209 | -0.2737 | No |
| 188 | DCAF1 | DDB1 and CUL4 associated factor 1 [Source:HGNC Symbol;Acc:HGNC:30911] | 8765 | -0.213 | -0.2767 | No |
| 189 | APEX1 | apurinic/apyrimidinic endodeoxyribonuclease 1 [Source:HGNC Symbol;Acc:HGNC:587] | 8767 | -0.213 | -0.2748 | No |
| 190 | RBPJ | recombination signal binding protein for immunoglobulin kappa J region [Source:HGNC Symbol;Acc:HGNC:5724] | 8800 | -0.217 | -0.2752 | No |
| 191 | TAF11 | TATA-box binding protein associated factor 11 [Source:HGNC Symbol;Acc:HGNC:11544] | 8860 | -0.222 | -0.2778 | No |
| 192 | TBX6 | T-box transcription factor 6 [Source:HGNC Symbol;Acc:HGNC:11605] | 8891 | -0.224 | -0.2781 | No |
| 193 | CHD4 | chromodomain helicase DNA binding protein 4 [Source:HGNC Symbol;Acc:HGNC:1919] | 8917 | -0.226 | -0.2779 | No |
| 194 | DAXX | death domain associated protein [Source:HGNC Symbol;Acc:HGNC:2681] | 8926 | -0.226 | -0.2764 | No |
| 195 | SIRT1 | sirtuin 1 [Source:HGNC Symbol;Acc:HGNC:14929] | 8947 | -0.227 | -0.2758 | No |
| 196 | SPI1 | Spi-1 proto-oncogene [Source:HGNC Symbol;Acc:HGNC:11241] | 9012 | -0.232 | -0.2787 | No |
| 197 | SMAD4 | SMAD family member 4 [Source:HGNC Symbol;Acc:HGNC:6770] | 9035 | -0.235 | -0.2782 | No |
| 198 | HAND1 | heart and neural crest derivatives expressed 1 [Source:HGNC Symbol;Acc:HGNC:4807] | 9082 | -0.238 | -0.2796 | No |
| 199 | FKBP4 | FKBP prolyl isomerase 4 [Source:HGNC Symbol;Acc:HGNC:3720] | 9205 | -0.248 | -0.2869 | No |
| 200 | LCOR | ligand dependent nuclear receptor corepressor [Source:HGNC Symbol;Acc:HGNC:29503] | 9227 | -0.250 | -0.2862 | No |
| 201 | BCL10 | BCL10 immune signaling adaptor [Source:HGNC Symbol;Acc:HGNC:989] | 9248 | -0.252 | -0.2854 | No |
| 202 | AHR | aryl hydrocarbon receptor [Source:HGNC Symbol;Acc:HGNC:348] | 9263 | -0.253 | -0.2841 | No |
| 203 | NCOA1 | nuclear receptor coactivator 1 [Source:HGNC Symbol;Acc:HGNC:7668] | 9270 | -0.253 | -0.2822 | No |
| 204 | POU4F1 | POU class 4 homeobox 1 [Source:HGNC Symbol;Acc:HGNC:9218] | 9300 | -0.256 | -0.2820 | No |
| 205 | HDAC2 | histone deacetylase 2 [Source:HGNC Symbol;Acc:HGNC:4853] | 9359 | -0.260 | -0.2842 | No |
| 206 | CRX | cone-rod homeobox [Source:HGNC Symbol;Acc:HGNC:2383] | 9397 | -0.263 | -0.2846 | No |
| 207 | PAGR1 | PAXIP1 associated glutamate rich protein 1 [Source:HGNC Symbol;Acc:HGNC:28707] | 9401 | -0.264 | -0.2823 | No |
| 208 | ARNT | aryl hydrocarbon receptor nuclear translocator [Source:HGNC Symbol;Acc:HGNC:700] | 9427 | -0.267 | -0.2818 | No |
| 209 | GTF2H1 | general transcription factor IIH subunit 1 [Source:HGNC Symbol;Acc:HGNC:4655] | 9455 | -0.269 | -0.2814 | No |
| 210 | NKX2-1 | NK2 homeobox 1 [Source:HGNC Symbol;Acc:HGNC:11825] | 9480 | -0.272 | -0.2807 | No |
| 211 | PRDM13 | PR/SET domain 13 [Source:HGNC Symbol;Acc:HGNC:13998] | 9484 | -0.273 | -0.2783 | No |
| 212 | DDIT3 | DNA damage inducible transcript 3 [Source:HGNC Symbol;Acc:HGNC:2726] | 9551 | -0.278 | -0.2809 | No |
| 213 | TACC2 | transforming acidic coiled-coil containing protein 2 [Source:HGNC Symbol;Acc:HGNC:11523] | 9581 | -0.281 | -0.2805 | No |
| 214 | PRKDC | "protein kinase, DNA-activated, catalytic subunit [Source:HGNC Symbol;Acc:HGNC:9413]" | 9632 | -0.286 | -0.2818 | No |
| 215 | TACC1 | transforming acidic coiled-coil containing protein 1 [Source:HGNC Symbol;Acc:HGNC:11522] | 9690 | -0.290 | -0.2836 | No |
| 216 | DDRGK1 | DDRGK domain containing 1 [Source:HGNC Symbol;Acc:HGNC:16110] | 9741 | -0.296 | -0.2847 | No |
| 217 | ETS1 | "ETS proto-oncogene 1, transcription factor [Source:HGNC Symbol;Acc:HGNC:3488]" | 9795 | -0.301 | -0.2861 | No |
| 218 | NKX2-5 | NK2 homeobox 5 [Source:HGNC Symbol;Acc:HGNC:2488] | 9875 | -0.309 | -0.2894 | No |
| 219 | MLXIPL | MLX interacting protein like [Source:HGNC Symbol;Acc:HGNC:12744] | 9882 | -0.310 | -0.2869 | No |
| 220 | FOXH1 | forkhead box H1 [Source:HGNC Symbol;Acc:HGNC:3814] | 9884 | -0.310 | -0.2841 | No |
| 221 | TRIM24 | tripartite motif containing 24 [Source:HGNC Symbol;Acc:HGNC:11812] | 9927 | -0.314 | -0.2844 | No |
| 222 | HMGA1 | high mobility group AT-hook 1 [Source:HGNC Symbol;Acc:HGNC:5010] | 9966 | -0.318 | -0.2844 | No |
| 223 | SMARCA4 | "SWI/SNF related, matrix associated, actin dependent regulator of chromatin, subfamily a, member 4 [Source:HGNC Symbol;Acc:HGNC:11100]" | 10009 | -0.322 | -0.2847 | No |
| 224 | TBXT | T-box transcription factor T [Source:HGNC Symbol;Acc:HGNC:11515] | 10034 | -0.325 | -0.2835 | No |
| 225 | KDM4C | lysine demethylase 4C [Source:HGNC Symbol;Acc:HGNC:17071] | 10040 | -0.325 | -0.2808 | No |
| 226 | DNAJA1 | DnaJ heat shock protein family (Hsp40) member A1 [Source:HGNC Symbol;Acc:HGNC:5229] | 10041 | -0.325 | -0.2777 | No |
| 227 | SMARCB1 | "SWI/SNF related, matrix associated, actin dependent regulator of chromatin, subfamily b, member 1 [Source:HGNC Symbol;Acc:HGNC:11103]" | 10117 | -0.333 | -0.2805 | No |
| 228 | CTBP1 | C-terminal binding protein 1 [Source:HGNC Symbol;Acc:HGNC:2494] | 10188 | -0.339 | -0.2828 | No |
| 229 | ACTN4 | actinin alpha 4 [Source:HGNC Symbol;Acc:HGNC:166] | 10472 | -0.370 | -0.3018 | No |
| 230 | PPARGC1A | PPARG coactivator 1 alpha [Source:HGNC Symbol;Acc:HGNC:9237] | 10504 | -0.374 | -0.3007 | No |
| 231 | MTA2 | metastasis associated 1 family member 2 [Source:HGNC Symbol;Acc:HGNC:7411] | 10600 | -0.383 | -0.3046 | Yes |
| 232 | EXOSC9 | exosome component 9 [Source:HGNC Symbol;Acc:HGNC:9137] | 10606 | -0.384 | -0.3013 | Yes |
| 233 | DHX9 | DExH-box helicase 9 [Source:HGNC Symbol;Acc:HGNC:2750] | 10627 | -0.386 | -0.2992 | Yes |
| 234 | FOS | "Fos proto-oncogene, AP-1 transcription factor subunit [Source:HGNC Symbol;Acc:HGNC:3796]" | 10640 | -0.387 | -0.2965 | Yes |
| 235 | NOTCH2 | notch receptor 2 [Source:HGNC Symbol;Acc:HGNC:7882] | 10720 | -0.395 | -0.2990 | Yes |
| 236 | BEX1 | brain expressed X-linked 1 [Source:HGNC Symbol;Acc:HGNC:1036] | 10772 | -0.402 | -0.2992 | Yes |
| 237 | SOX17 | SRY-box transcription factor 17 [Source:HGNC Symbol;Acc:HGNC:18122] | 10843 | -0.410 | -0.3009 | Yes |
| 238 | CREBBP | CREB binding protein [Source:HGNC Symbol;Acc:HGNC:2348] | 10881 | -0.414 | -0.2999 | Yes |
| 239 | GRIP1 | glutamate receptor interacting protein 1 [Source:HGNC Symbol;Acc:HGNC:18708] | 10949 | -0.424 | -0.3012 | Yes |
| 240 | GATA1 | GATA binding protein 1 [Source:HGNC Symbol;Acc:HGNC:4170] | 10958 | -0.425 | -0.2978 | Yes |
| 241 | RNF25 | ring finger protein 25 [Source:HGNC Symbol;Acc:HGNC:14662] | 11015 | -0.432 | -0.2981 | Yes |
| 242 | ETS2 | "ETS proto-oncogene 2, transcription factor [Source:HGNC Symbol;Acc:HGNC:3489]" | 11057 | -0.439 | -0.2972 | Yes |
| 243 | GATA2 | GATA binding protein 2 [Source:HGNC Symbol;Acc:HGNC:4171] | 11058 | -0.439 | -0.2930 | Yes |
| 244 | STK4 | serine/threonine kinase 4 [Source:HGNC Symbol;Acc:HGNC:11408] | 11074 | -0.441 | -0.2900 | Yes |
| 245 | PPARA | peroxisome proliferator activated receptor alpha [Source:HGNC Symbol;Acc:HGNC:9232] | 11110 | -0.445 | -0.2886 | Yes |
| 246 | TBP | TATA-box binding protein [Source:HGNC Symbol;Acc:HGNC:11588] | 11138 | -0.448 | -0.2865 | Yes |
| 247 | MED1 | mediator complex subunit 1 [Source:HGNC Symbol;Acc:HGNC:9234] | 11146 | -0.450 | -0.2827 | Yes |
| 248 | MEF2D | myocyte enhancer factor 2D [Source:HGNC Symbol;Acc:HGNC:6997] | 11149 | -0.451 | -0.2786 | Yes |
| 249 | GSK3B | glycogen synthase kinase 3 beta [Source:HGNC Symbol;Acc:HGNC:4617] | 11157 | -0.451 | -0.2749 | Yes |
| 250 | HSF1 | heat shock transcription factor 1 [Source:HGNC Symbol;Acc:HGNC:5224] | 11189 | -0.456 | -0.2730 | Yes |
| 251 | CRY1 | cryptochrome circadian regulator 1 [Source:HGNC Symbol;Acc:HGNC:2384] | 11247 | -0.463 | -0.2731 | Yes |
| 252 | PTPN2 | protein tyrosine phosphatase non-receptor type 2 [Source:HGNC Symbol;Acc:HGNC:9650] | 11270 | -0.467 | -0.2704 | Yes |
| 253 | PIAS2 | protein inhibitor of activated STAT 2 [Source:HGNC Symbol;Acc:HGNC:17311] | 11288 | -0.470 | -0.2673 | Yes |
| 254 | PPARD | peroxisome proliferator activated receptor delta [Source:HGNC Symbol;Acc:HGNC:9235] | 11301 | -0.471 | -0.2638 | Yes |
| 255 | TMF1 | TATA element modulatory factor 1 [Source:HGNC Symbol;Acc:HGNC:11870] | 11328 | -0.475 | -0.2613 | Yes |
| 256 | NKX3-1 | NK3 homeobox 1 [Source:HGNC Symbol;Acc:HGNC:7838] | 11348 | -0.478 | -0.2583 | Yes |
| 257 | SETD6 | "SET domain containing 6, protein lysine methyltransferase [Source:HGNC Symbol;Acc:HGNC:26116]" | 11467 | -0.498 | -0.2629 | Yes |
| 258 | BRD8 | bromodomain containing 8 [Source:HGNC Symbol;Acc:HGNC:19874] | 11557 | -0.508 | -0.2651 | Yes |
| 259 | ZBTB7A | zinc finger and BTB domain containing 7A [Source:HGNC Symbol;Acc:HGNC:18078] | 11600 | -0.516 | -0.2636 | Yes |
| 260 | NR4A1 | nuclear receptor subfamily 4 group A member 1 [Source:HGNC Symbol;Acc:HGNC:7980] | 11709 | -0.535 | -0.2671 | Yes |
| 261 | EP300 | E1A binding protein p300 [Source:HGNC Symbol;Acc:HGNC:3373] | 11741 | -0.540 | -0.2644 | Yes |
| 262 | EPAS1 | endothelial PAS domain protein 1 [Source:HGNC Symbol;Acc:HGNC:3374] | 11758 | -0.542 | -0.2605 | Yes |
| 263 | LATS1 | large tumor suppressor kinase 1 [Source:HGNC Symbol;Acc:HGNC:6514] | 11760 | -0.542 | -0.2554 | Yes |
| 264 | CTDP1 | CTD phosphatase subunit 1 [Source:HGNC Symbol;Acc:HGNC:2498] | 11805 | -0.550 | -0.2537 | Yes |
| 265 | HIF1A | hypoxia inducible factor 1 subunit alpha [Source:HGNC Symbol;Acc:HGNC:4910] | 11833 | -0.556 | -0.2506 | Yes |
| 266 | PDCD11 | programmed cell death 11 [Source:HGNC Symbol;Acc:HGNC:13408] | 11847 | -0.559 | -0.2463 | Yes |
| 267 | ID2 | inhibitor of DNA binding 2 [Source:HGNC Symbol;Acc:HGNC:5361] | 11852 | -0.560 | -0.2413 | Yes |
| 268 | TRIB1 | tribbles pseudokinase 1 [Source:HGNC Symbol;Acc:HGNC:16891] | 11924 | -0.577 | -0.2414 | Yes |
| 269 | TCERG1 | transcription elongation regulator 1 [Source:HGNC Symbol;Acc:HGNC:15630] | 11950 | -0.583 | -0.2378 | Yes |
| 270 | DDX54 | DEAD-box helicase 54 [Source:HGNC Symbol;Acc:HGNC:20084] | 11969 | -0.587 | -0.2337 | Yes |
| 271 | ATF4 | activating transcription factor 4 [Source:HGNC Symbol;Acc:HGNC:786] | 11999 | -0.593 | -0.2304 | Yes |
| 272 | SPEN | spen family transcriptional repressor [Source:HGNC Symbol;Acc:HGNC:17575] | 12108 | -0.622 | -0.2330 | Yes |
| 273 | ARNTL | aryl hydrocarbon receptor nuclear translocator like [Source:HGNC Symbol;Acc:HGNC:701] | 12120 | -0.625 | -0.2279 | Yes |
| 274 | HIRA | histone cell cycle regulator [Source:HGNC Symbol;Acc:HGNC:4916] | 12143 | -0.630 | -0.2237 | Yes |
| 275 | HMGB1 | high mobility group box 1 [Source:HGNC Symbol;Acc:HGNC:4983] | 12170 | -0.636 | -0.2197 | Yes |
| 276 | TEAD3 | TEA domain transcription factor 3 [Source:HGNC Symbol;Acc:HGNC:11716] | 12178 | -0.637 | -0.2142 | Yes |
| 277 | GATA3 | GATA binding protein 3 [Source:HGNC Symbol;Acc:HGNC:4172] | 12276 | -0.670 | -0.2155 | Yes |
| 278 | NR4A2 | nuclear receptor subfamily 4 group A member 2 [Source:HGNC Symbol;Acc:HGNC:7981] | 12368 | -0.698 | -0.2161 | Yes |
| 279 | REST | RE1 silencing transcription factor [Source:HGNC Symbol;Acc:HGNC:9966] | 12382 | -0.703 | -0.2105 | Yes |
| 280 | THRAP3 | thyroid hormone receptor associated protein 3 [Source:HGNC Symbol;Acc:HGNC:22964] | 12426 | -0.717 | -0.2071 | Yes |
| 281 | STRN | striatin [Source:HGNC Symbol;Acc:HGNC:11424] | 12429 | -0.717 | -0.2004 | Yes |
| 282 | NCOA3 | nuclear receptor coactivator 3 [Source:HGNC Symbol;Acc:HGNC:7670] | 12455 | -0.727 | -0.1955 | Yes |
| 283 | MED25 | mediator complex subunit 25 [Source:HGNC Symbol;Acc:HGNC:28845] | 12480 | -0.741 | -0.1903 | Yes |
| 284 | MED13 | mediator complex subunit 13 [Source:HGNC Symbol;Acc:HGNC:22474] | 12504 | -0.749 | -0.1850 | Yes |
| 285 | NPM1 | nucleophosmin 1 [Source:HGNC Symbol;Acc:HGNC:7910] | 12530 | -0.762 | -0.1798 | Yes |
| 286 | NR4A3 | nuclear receptor subfamily 4 group A member 3 [Source:HGNC Symbol;Acc:HGNC:7982] | 12543 | -0.769 | -0.1734 | Yes |
| 287 | SRF | serum response factor [Source:HGNC Symbol;Acc:HGNC:11291] | 12549 | -0.773 | -0.1665 | Yes |
| 288 | SP1 | Sp1 transcription factor [Source:HGNC Symbol;Acc:HGNC:11205] | 12557 | -0.777 | -0.1596 | Yes |
| 289 | BHLHE40 | basic helix-loop-helix family member e40 [Source:HGNC Symbol;Acc:HGNC:1046] | 12573 | -0.782 | -0.1534 | Yes |
| 290 | KDM5D | lysine demethylase 5D [Source:HGNC Symbol;Acc:HGNC:11115] | 12595 | -0.793 | -0.1475 | Yes |
| 291 | GABARAPL1 | GABA type A receptor associated protein like 1 [Source:HGNC Symbol;Acc:HGNC:4068] | 12616 | -0.808 | -0.1414 | Yes |
| 292 | MTDH | metadherin [Source:HGNC Symbol;Acc:HGNC:29608] | 12621 | -0.809 | -0.1340 | Yes |
| 293 | HES1 | hes family bHLH transcription factor 1 [Source:HGNC Symbol;Acc:HGNC:5192] | 12676 | -0.849 | -0.1302 | Yes |
| 294 | ARID5A | AT-rich interaction domain 5A [Source:HGNC Symbol;Acc:HGNC:17361] | 12705 | -0.867 | -0.1242 | Yes |
| 295 | ACTB | actin beta [Source:HGNC Symbol;Acc:HGNC:132] | 12757 | -0.920 | -0.1195 | Yes |
| 296 | MTA1 | metastasis associated 1 [Source:HGNC Symbol;Acc:HGNC:7410] | 12792 | -0.952 | -0.1132 | Yes |
| 297 | SETD1A | "SET domain containing 1A, histone lysine methyltransferase [Source:HGNC Symbol;Acc:HGNC:29010]" | 12793 | -0.954 | -0.1041 | Yes |
| 298 | CEBPB | CCAAT enhancer binding protein beta [Source:HGNC Symbol;Acc:HGNC:1834] | 12804 | -0.973 | -0.0956 | Yes |
| 299 | JUN | "Jun proto-oncogene, AP-1 transcription factor subunit [Source:HGNC Symbol;Acc:HGNC:6204]" | 12813 | -0.994 | -0.0868 | Yes |
| 300 | NFKBIA | NFKB inhibitor alpha [Source:HGNC Symbol;Acc:HGNC:7797] | 12817 | -1.001 | -0.0775 | Yes |
| 301 | DDX5 | DEAD-box helicase 5 [Source:HGNC Symbol;Acc:HGNC:2746] | 12834 | -1.038 | -0.0689 | Yes |
| 302 | TOB2 | "transducer of ERBB2, 2 [Source:HGNC Symbol;Acc:HGNC:11980]" | 12838 | -1.041 | -0.0593 | Yes |
| 303 | HIPK2 | homeodomain interacting protein kinase 2 [Source:HGNC Symbol;Acc:HGNC:14402] | 12842 | -1.045 | -0.0496 | Yes |
| 304 | KLF4 | Kruppel like factor 4 [Source:HGNC Symbol;Acc:HGNC:6348] | 12848 | -1.058 | -0.0399 | Yes |
| 305 | JUND | "JunD proto-oncogene, AP-1 transcription factor subunit [Source:HGNC Symbol;Acc:HGNC:6206]" | 12865 | -1.116 | -0.0305 | Yes |
| 306 | CTNNB1 | catenin beta 1 [Source:HGNC Symbol;Acc:HGNC:2514] | 12866 | -1.119 | -0.0199 | Yes |
| 307 | BAZ2A | bromodomain adjacent to zinc finger domain 2A [Source:HGNC Symbol;Acc:HGNC:962] | 12879 | -1.166 | -0.0098 | Yes |
| 308 | TAF1 | TATA-box binding protein associated factor 1 [Source:HGNC Symbol;Acc:HGNC:11535] | 12881 | -1.192 | 0.0015 | Yes |
Table: GSEA details [plain text format]

  

Fig 2: GOMF\_RNA\_POLYMERASE\_II\_SPECIFIC\_DNA\_BINDING\_TRANSCRIPTION\_FACTOR\_BINDING      
 Blue-Pink O' Gram in the Space of the Analyzed GeneSet

  

Fig 3: GOMF\_RNA\_POLYMERASE\_II\_SPECIFIC\_DNA\_BINDING\_TRANSCRIPTION\_FACTOR\_BINDING: Random ES distribution      
 Gene set null distribution of ES for **GOMF\_RNA\_POLYMERASE\_II\_SPECIFIC\_DNA\_BINDING\_TRANSCRIPTION\_FACTOR\_BINDING**
